# Insights into the Prostanoid Pathway in the Ovary Development of the Penaeid Shrimp *Penaeus monodon*

**DOI:** 10.1371/journal.pone.0076934

**Published:** 2013-10-08

**Authors:** Wananit Wimuttisuk, Punsa Tobwor, Pacharawan Deenarn, Kannawat Danwisetkanjana, Decha Pinkaew, Kanyawim Kirtikara, Vanicha Vichai

**Affiliations:** National Center for Genetic Engineering and Biotechnology (BIOTEC), National Science and Technology Development Agency, Khlong Luang, Pathum Thani, Thailand; John Hunter Hospital, Australia

## Abstract

The prostanoid pathway converts polyunsaturated fatty acids (PUFAs) into bioactive lipid mediators, including prostaglandins, thromboxanes and prostacyclins, all of which play vital roles in the immune and reproductive systems in most animal phyla. In crustaceans, PUFAs and prostaglandins have been detected and often associated with female reproductive maturation. However, the presence of prostanoid biosynthesis genes remained in question in these species. In this study, we outlined the prostanoid pathway in the black tiger shrimp *Penaeus monodon* based on the amplification of nine prostanoid biosynthesis genes: *cytosolic phospholipase A2, hematopoietic prostaglandin D synthase, glutathione-dependent prostaglandin D synthase, prostaglandin E synthase 1, prostaglandin E synthase 2, prostaglandin E synthase 3, prostaglandin F synthase*, *thromboxane A synthase* and *cyclooxygenase*. TBLASTX analysis confirmed the identities of these genes with 51-99% sequence identities to their closest homologs. In addition, prostaglandin F_2α_ (PGF_2α_), which is a product of the prostaglandin F synthase enzyme, was detected for the first time in *P. monodon* ovaries along with the previously identified PUFAs and prostaglandin E_2_ (PGE_2_) using RP-HPLC and mass-spectrometry. The prostaglandin synthase activity was also observed in shrimp ovary homogenates using *in vitro* activity assay. When prostaglandin biosynthesis was examined in different stages of shrimp ovaries, we found that the amounts of *prostaglandin F synthase* gene transcripts and PGF_2α_ decreased as the ovaries matured. These findings not only indicate the presence of a functional prostanoid pathway in penaeid shrimp, but also suggest a possible role of the PGF_2α_ biosynthesis in shrimp ovarian development.

## Introduction

Prostanoids are oxygenated derivatives of C-20 polyunsaturated fatty acids (PUFAs) that play active roles in inflammation, immune response, cardiovascular control and reproduction in most animals [1-3]. These PUFAs, which serve as precursors of the prostanoid pathway, include arachidonic acid (AA), eicosapentaenoic acid (EPA) and docosahexaenoic acid (DHA). The prostanoid pathway begins with the enzyme phospholipase A_2_, which releases AA from the phospholipids of cellular and intracellular membranes [3]. The released AA is then cyclized and subsequently reduced by the cyclooxygenase (COX) enzyme to form prostaglandin G_2_ (PGG_2_) and prostaglandin H_2_ (PGH_2_), respectively [4,5]. Downstream enzymes, including prostaglandin and thromboxane synthases, later convert PGH_2_ to prostanoids, such as prostaglandins, prostacyclins and thromboxanes, which serve as signaling molecules in various physiological responses [3,6].

The presence of PUFAs and prostaglandins in crustaceans has long been the focus of aquaculture research. All three prostanoid precursors (AA, EPA and DHA) have been identified in the Chinese prawn *Penaeus chinensis* [7], the Pacific white shrimp *Litopenaeus vannamei* [8], the green tiger prawn *Penaeus semisulcatus* [9], the kuruma prawn *Marsupenaeus japonicus* [10,11], the banana shrimp *Penaeus merguiensis* [12] and *Penaeus monodon* [13,14]. In addition, EPA has been identified in the common littoral crab *Carcinus maenas* [15] and the Atlantic blue crab *Callinectes sapidus* [16], while DHA has been detected in the crayfish *Procambarus clarkii* [17]. On the other hand, prostaglandin E_2_ (PGE_2_) and prostaglandin F_2α_ (PGF_2α_) have been identified in *M. japonicus* [10] and the Florida crayfish *Procambarus paeninsulanus* [18,19]. Prostaglandin D_2_ (PGD_2_), PGE_2_ and PGF_2α_ have been detected in the fresh water field crab *Oziotelphusa senex senex* [20], while PGE_2_, thromboxane B2 (TXB_2_) and 6-keto-PGF_1α_ have been reported in *C. maenas* [15]. In addition, PGE_2_ has been identified in hemolymph, muscle and ovary of domesticated *P. monodon* [21], but the presence of PGF_2α_ has not been reported in this species.

In crustaceans, one of the more prominent roles of prostanoids is the regulation of female reproductive maturation. For instance, the production of PGE_2_ and PGF_2α_ is positively correlated with ovarian maturation in *P. paeninsulanus* [18] and *O. senex senex* [20]. Furthermore, injection of PGE_2_ and PGF_2α_ into ovaries of *O. senex senex* significantly increased the number and the diameter of the oocytes in a dose-dependent manner [20]. In domesticated *P. monodon*, the amounts of PGE_2_ in ovaries and haemolymph increased along with developing ovary stages [21]. However, the correlation between prostaglandins and crustacean ovary development may be species specific, as the amounts of PGE_2_ and PGF_2α_ were highest in ovaries stage I and continued to decrease until stage IV in *M. japonicus* [10]. Nevertheless, these findings suggest a possible involvement of the prostanoid biosynthesis in crustacean female reproductive system.

Although the production of prostanoids and their precursors is well-established in most crustaceans, prostanoid biosynthesis genes in these species are poorly characterized. Thus far, the only crustacean with a fully constructed prostanoid pathway is the fresh water flea *Daphnia pulex*, whose annotated genome sequence revealed nine prostanoid biosynthesis genes: *cytosolic phospholipase A2* (*cPLA2*)*, COX, prostaglandin D2 synthase A, prostaglandin D2 synthase B, prostaglandin E2 synthase* (*PGES*)*, carbonyl reductase 1, thromboxane A* and *thromboxane B*, and two prostanoid receptors *prostanoid receptor EP4 isoform A* and *B* [22]. In marine crustaceans, *COX* genes have been identified in *Gammarus* spp. and *Caprella* spp. [23], while *PGES* genes have been characterized in *L. vannamei* [24], the American lobster *Homarus americanus* (Accession: MGID155886 from the Marine Genomic Project) [24] and the sea lice *Lepeophtheirus salmonis* and *Caligus rogercresseyi* [25].

Due to the roles of prostanoids in the reproductive system in most crustaceans, the characterization of the prostanoid pathway in economically valuable organisms, such as penaeid shrimp, is essential for both scientific gain and potential applications in aquaculture practice. In this study, we propose a scheme for the *P. monodon* prostanoid pathway based on the identification of eight *P. monodon* prostanoid biosynthesis genes, the detection of lipid precursors and prostaglandins, and the detection of prostaglandin synthase activity. The correlations observed among gene transcription, prostaglandin production, and ovarian maturation also suggest that prostaglandin biosynthesis may be involved in the regulation of the *P. monodon* female reproductive system.

## Results

### Identification of *P. monodon* prostanoid biosynthesis genes

Based on available EST sequences from The Black Tiger Shrimp EST Project [26] and the Marine Genomics Project [24], short fragments of *P. monodon* prostanoid biosynthesis genes were amplified from shrimp ovary cDNA. RACE-PCR was used to obtain full-length gene sequences, resulting in the identification of nine putative *P. monodon* prostanoid biosynthesis genes: *cytosolic phospholipase A2* (*PmcPLA2*)*, hematopoietic prostaglandin D synthase* (*PmhPGDS*)*, glutathione-dependent prostaglandin D synthase* (*PmgPGDS*)*, prostaglandin E synthase 1* (*PmPGES1*)*, prostaglandin E synthase 2* (*PmPGES2*)*, prostaglandin E synthase 3* (*PmPGES3*)*, prostaglandin F synthase* (*PmPGFS*), *thromboxane A synthase* (*PmTBXAS*), and *cyclooxygenase* (*PmCOX*). These gene sequences were then analyzed by TBLASTX, revealing 51-99% sequence identities of the predicted *P. monodon* enzymes when compared with their closest homologs (Table 1).

**Table 1:** TBLASTX analyses of *P. monodon* prostanoid biosynthesis genes.

**Genes**	***Pm* accession #**	**AA**	**Matched accession # (Reference species)**	**Identity**	**E-value**
*cPLA2*	JN003878	998	NM_001081843 (*Equus caballus*)	60%	2e-180
*hPGDS*	JN003879	203	HM231278 (*Eriocheir sinensis*)	99%	5e-37
*gPGDS*	JN003880	153	XM_003694283 (*Apis florea*)	79%	4e-23
*PGES3*	JN003881	164	JF806619 (*Litopenaeus vannamei*)	51%	2e-108
*PGES1*	JN003882	145	JQ917473 (*Nilaparvata lugens*)	76%	7e-35
*PGES2*	JN003883	338	XM_003706983 (*Megachile rotundata*)	89%	4e-108
*PGFS*	JN003884	317	JX513906 (*Reticulitermes flavipes*)	98%	3e-67
*TBXAS*	JN003885	533	XM_004069858 (*Oryzias latipes*)	60%	8e-62
*COX*	KF501342	614	GQ180796 (*Gammarus* *sp.*)	91%	0.0

### Analysis of conserved residues and domains in *PmPGES1, PmPGES2, PmPGES3* and *PmPGFS* genes

The putative *P. monodon* prostanoid biosynthesis genes were submitted to the Conserved Domain Architecture Retrieval Tool (CDART) for protein domain prediction, revealing that the *P. monodon* proteins contain the same domain types and positions as the prostanoid enzymes found in other species (Figure S1). At this point, *PmPGES* and *PmPGFS* were examined in more details, as these genes are likely to be responsible for the biosynthesis of PGE_2_ and PGF_2α,_ which has been shown to affect ovarian development in other crustaceans [10,18,20].

**Figure 1:**
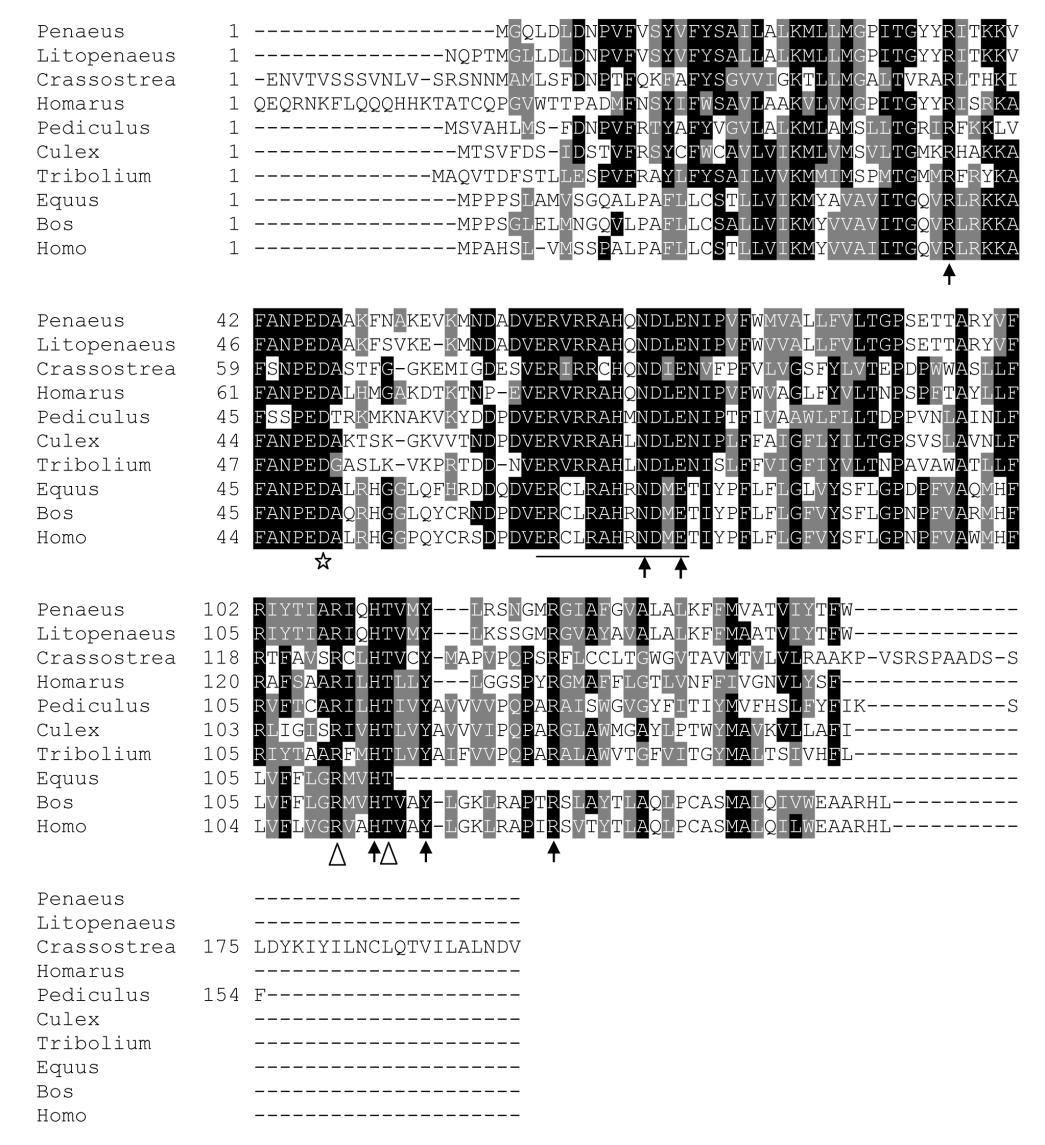
Mapping of essential residues in the predicted *P. monodon* PGES1 enzyme. Multiple sequence alignments of *P. monodon* PGES1 and their homologs showed a conserved residue for the MAPEG family (white star), catalytic residues that interact with PGH_2_ (white arrow head), essential residues for H-bonding to GSH (black arrows) and consensus sequence required for oxygenation product (underline). Genus and species used in this alignment are abbreviated as followed: *Penaeus* − *P. monodon*, Litopenaeus − *L. vannamei*, *Crassostrea* − *Crassostrea virginica*, Homarus − *H. americanus*, *Pediculus* − *Pediculus humanus corporis*, *Culex* − *Culex quinquefasciatus*, *Tribolium* − *Tribolium castaneum*, Equus − *Equus caballus*, Bos − *Bos taurus* and Homo − *Homo sapiens*.

In *P. monodon*, three isoforms of *PmPGES* have been cloned and characterized, namely *PmPGES1*, *PmPGES2* and *PmPGES3*, which have corresponding isoforms in mammals. *PGES1* or *membrane-associated prostaglandin E synthase 1* is a member of the Membrane-Associated Protein involved in Eicosanoid and Glutathione metabolism (MAPEG) [27,28]. *PGES1* requires glutathione for its enzymatic function, which involves converting PGH_2_ to PGE_2_ [27]. Multiple sequence alignment of PmPGES1 revealed the conservation of most catalytic residues. For example, D47, which is highly conserved in the PGES1 subgroup of MAPEG, was found in PmPGES1 (Figure 1, star) [29]. In addition, key catalytic residues that interact with PGH_2_ (R108 and T112 - Figure 1, white arrow heads) as well as glutathione (R36, N72, E75, H111, Y115 and R122 - Figure 1, black arrows) were conserved in PmPGES1 [29-31]. Lastly, PmPGES1 also contains the consensus sequence^64^ERXXXAXXNXX^75^ E required for oxygenation product formation (Figure 1, underlined) [31], confirming that PmPGES1 possesses all the necessary residues for its enzymatic function.

**Figure 2:**
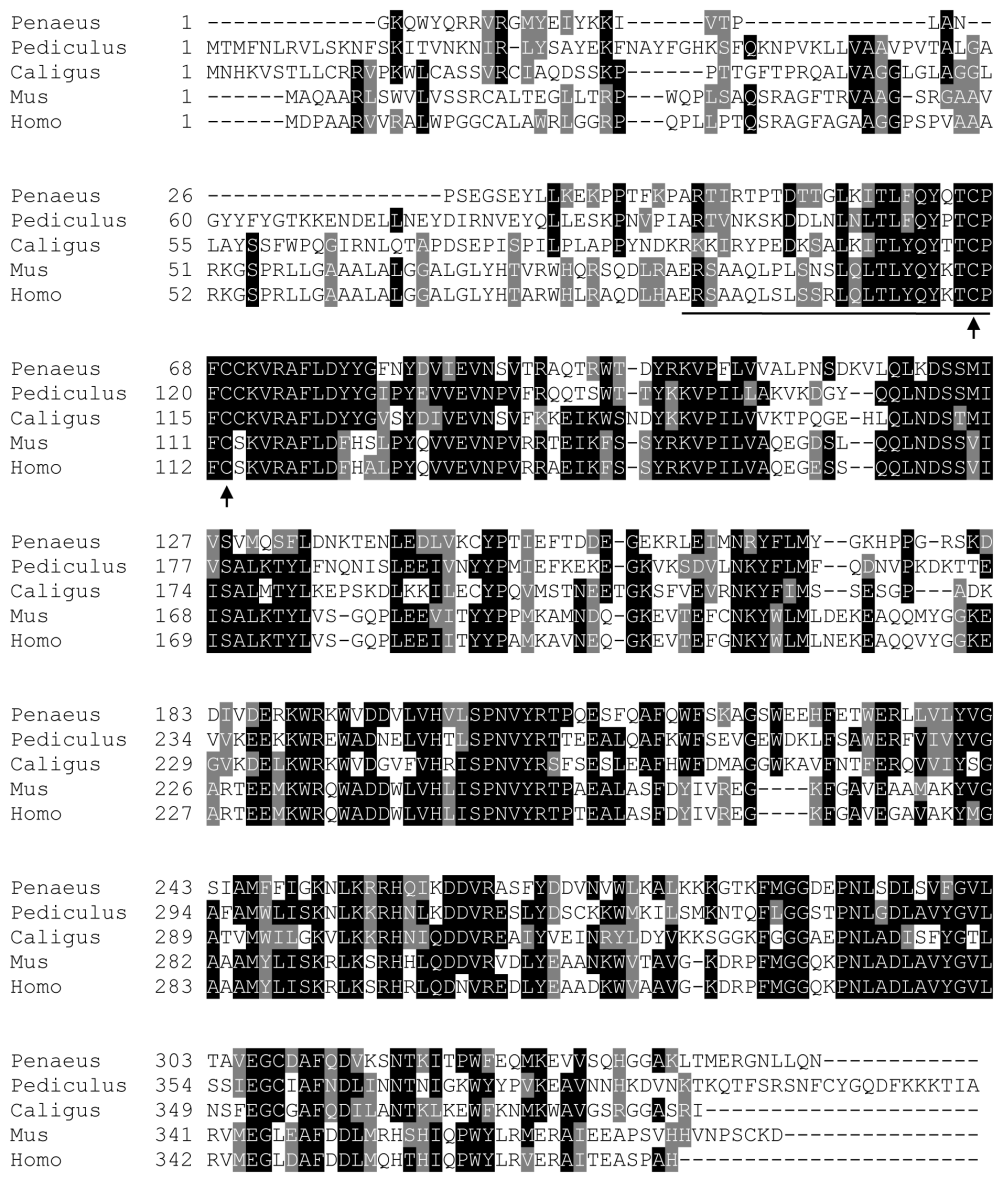
Mapping of essential residues in the predicted *P. monodon* PGES2 enzyme. Multiple sequence alignments of *P. monodon* PGES2 protein and their homologs were performed, revealing conserved Cys residues at the active site (black arrows) and the N-terminal sequence of the mature enzyme (underline). Genus and species used in this alignment are abbreviated as followed: *Penaeus* − *P. monodon*, *Pediculus* − *P. humanus corporis*, Caligus − *Caligus rogercresseyi*, Mus − *Mus musculus* and Homo − *H. sapiens*.

*PGES2* or *membrane-associated prostaglandin E synthase 2* is a Golgi membrane-associated pre-protein that requires spontaneous cleavage of the N-terminal hydrophobic domain to become a mature cytosolic enzyme [32,33]. The catalytic domain of PGES2 is a glutathione/thioredoxin-like domain that can be activated by various thiol reducing reagents [34]. Sequence alignment of PmPGES2 revealed a conserved ^66^C-X-X- ^69^C (Figure 2, black arrow), which corresponded to^110^C-X-X-^113^ C catalytic triad in the human PGES2 active site [35]. A conserved N-terminal hydrophobic domain (Figure 2, underlined) was also identified in PmPGES2, consistent with N-terminal cleavage to generate the mature enzyme.

**Figure 3:**
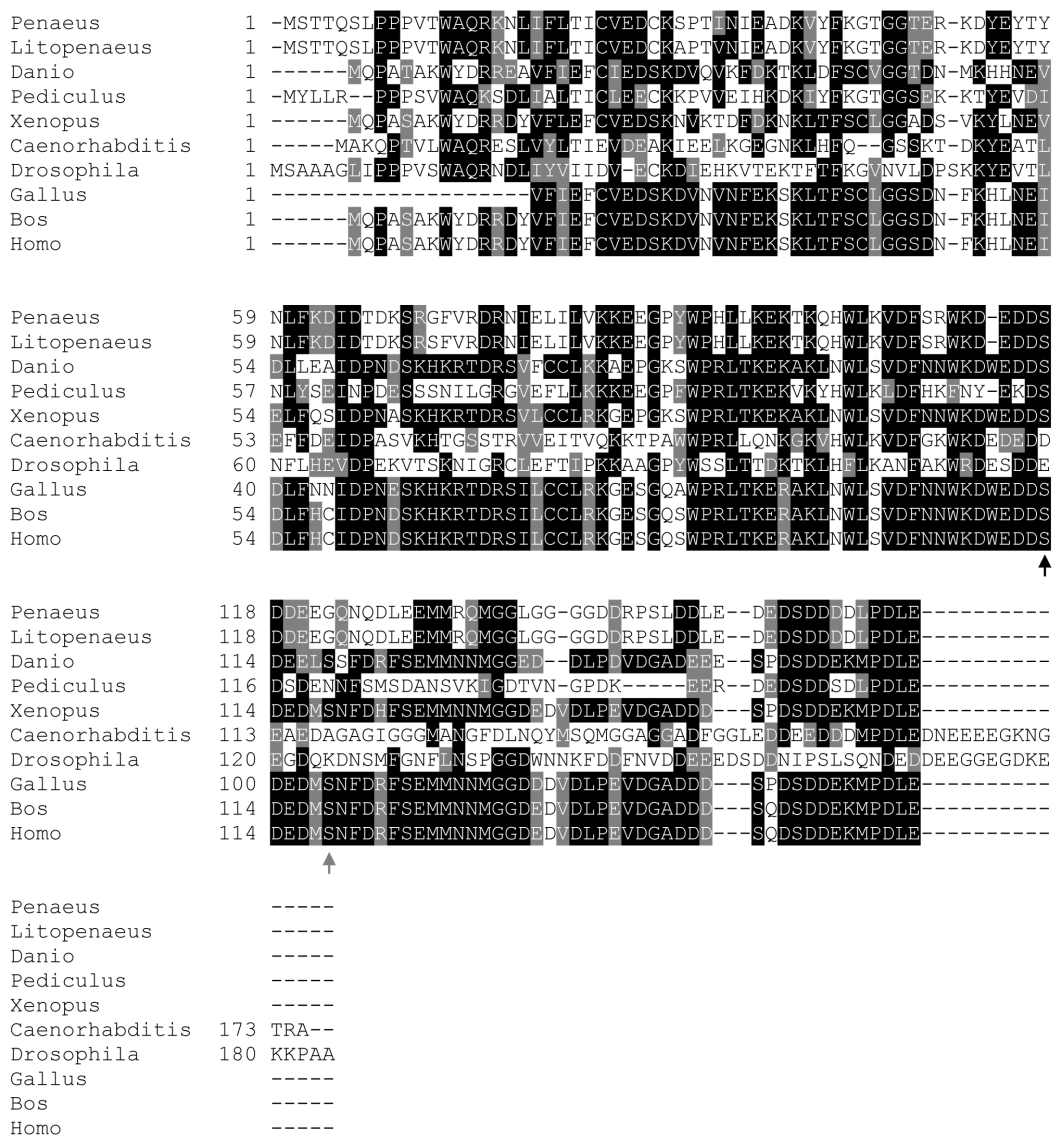
Mapping of essential residues in the predicted *P. monodon* PGES3 enzyme. Multiple sequence alignments of *P. monodon* PGES3 protein and their homologs reveal one conserved serine residue for the CKII phosphorylation site (black arrow), while the other phosphorylation site was not conserved (gray arrow). Genus and species used in this alignment are abbreviated as followed: *Penaeus* − *P. monodon*, Litopenaeus − *L. vannamei*, Danio − *Danio rerio*, *Pediculus* − *P. humanus corporis*, *Xenopus* − *Xenopus laevis*, *Caenorhabditis* − *Caenorhabditis elegans*, Drosophila − *Drosophila melanogaster*, Gallus − *Gallus gallus*, Bos − *B. taurus* and Homo − *H. sapiens*.

*PGES3* or *cytosolic prostaglandin E synthase* (*cPGES*) is a 23 kDa GSH-requiring enzyme that was originally termed p23 based on its initial characterization as a co-chaperone of heat shock protein 90 [28,36]. Unlike the membrane-bound PGES1 and PGES2, PGES3 is a cytosolic, glutathione-requiring enzyme that interacts with casein kinase II (CKII) and Hsp90 [36,37]. In human PGES3, two serine residues (S113 and S118 − Figure 3, arrows) are phosphorylated by CKII to increase PGES3 enzymatic activity. However, only the putative N-terminal phosphorylated serine is conserved in PmPGES3 (Figure 3, black arrow) [37]. CDART prediction of the putative PmPGES3 enzyme revealed the presence of an alpha crystallin-Hsps-p23 like super family domain, which is the same domain found in all PGES3 homologs (Figure S1), further confirming the identity of the *PmPGES*3 gene.

**Figure 4:**
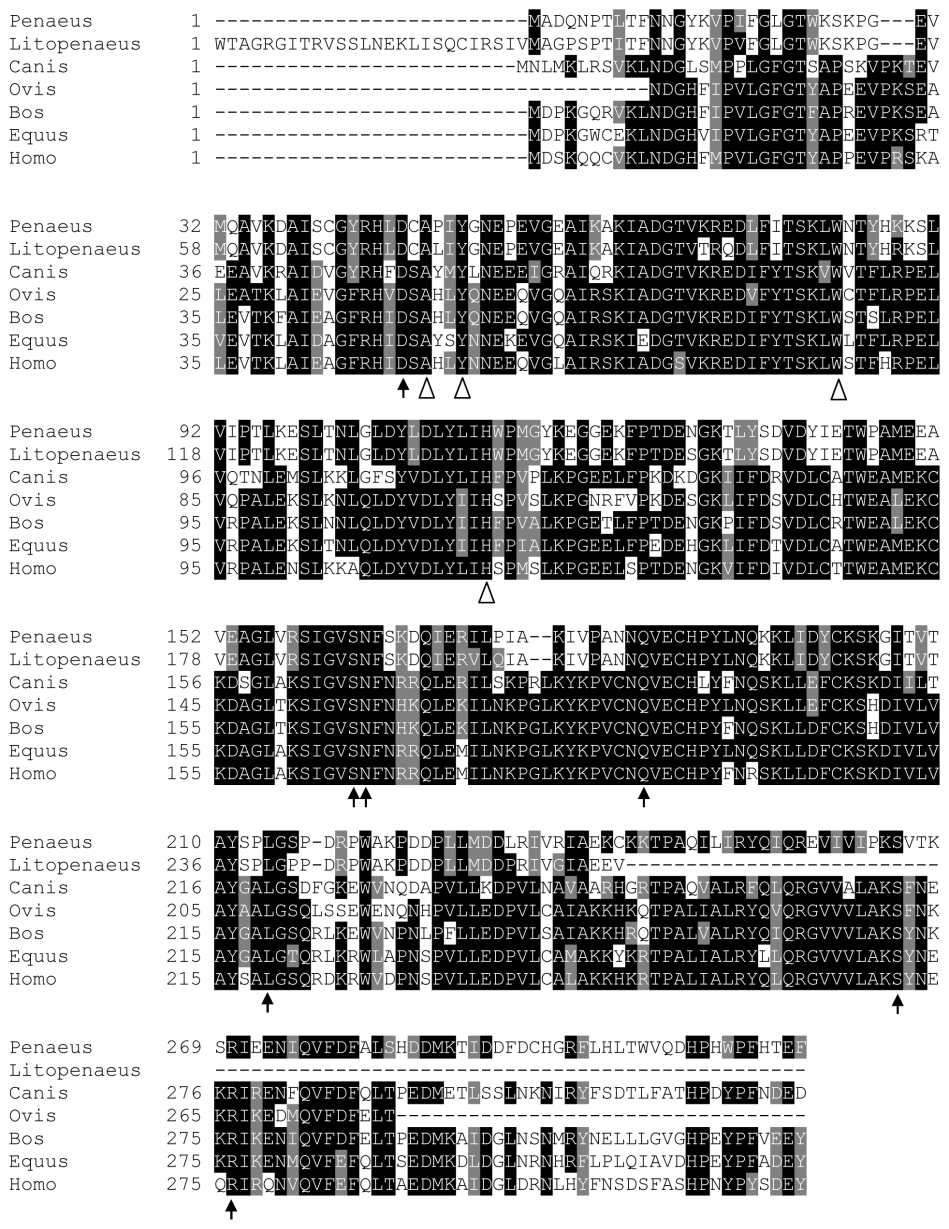
Mapping of essential residues in the predicted *P. monodon* PGFS enzyme. Multiple sequence alignments of *P. monodon* PGFS protein and their homologs were performed, revealing residues that are important for substrate (black arrows) and the NADP^+^ cofactor (white arrow head) binding. Genus and species used in this alignment are abbreviated as followed: *Penaeus* − *P. monodon*, Litopenaeus − *L. vannamei*, Canis − *Canis lupus familiaris*, Ovis − *Ovis aries*, Bos − *B. taurus*, Equus − *Equus caballus* and Homo − *H. sapiens*.

Lastly, *PGFS* encodes a bifunctional enzyme in the aldo-keto reductase (AKR) superfamily that converts PGD_2_ and PGH_2_ to (5*Z*,13*E*)-(15*S*)-9α,11β,15-trihydroxyprosta-5,13-dien-l-oic acid (9α,11β-PGF_2_) and PGF_2α_, respectively [38-40]. CDART analysis revealed that PmPGFS contains the domain in the aldo-keto reductase superfamily (Figure S1), which is characteristic of PGFS enzymes [41]. Multiple sequence alignment also indicated that residues required for substrate binding site (D49, S165, N166, Q189, L218, S270, R275 - Figure 4, black arrows) and NADP^+^ cofactor binding site (A51, Y54, W85, H116 - Figure 4, white arrow heads) are also conserved in PmPGFS [42].

### RP-HPLC and mass spectrometry analysis of PUFAs and prostaglandins in shrimp ovaries

**Figure 5:**
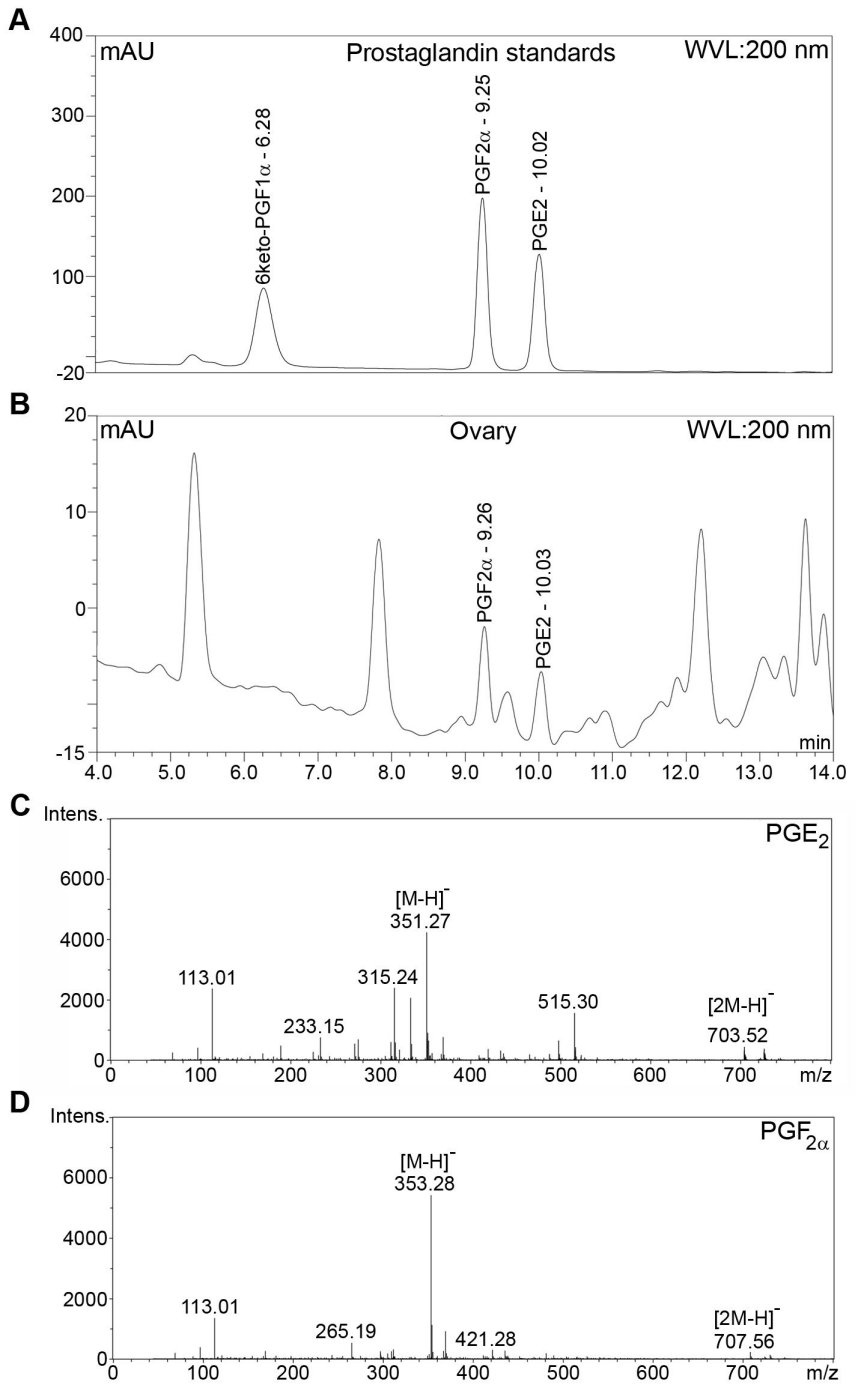
HPLC analysis and mass spectra of the prostaglandins in shrimp ovary extract. Ovaries from 5 wild broodstock were homogenized in HBSS, pooled together and incubated at 28 °C, 200 rpm for 1 h. The homogenate was extracted and analyzed by RP-HPLC and mass spectrometry as described in Materials and Methods. RP-HPLC elution profiles at 200 nm wavelength of commercially available prostaglandin standards (A) and prostaglandins found in ovary homogenate (B). Subsequent mass spectrometry analysis of PG in ovary homogenate yielded mass spectra of PGE_2_ (C) and PGF_2α_ (D).

Once the prostanoid biosynthesis genes had been identified, chemical analysis was performed to detect the presence of corresponding prostanoids in wild *P. monodon*. Stage IV shrimp ovaries from five broodstock were pooled, homogenized and subjected to solvent extraction. Subsequent RP-HPLC analysis led to the detection of PGF_2α_ and PGE_2_ as two small peaks that eluted at 9.26 and 10.03 minutes, respectively (Figure 5A and 5B). The identities of these prostaglandins were later confirmed by mass analysis (Figure 5C and 5D). In addition, three prostanoid precursors EPA, DHA and AA were detected with elution times of 30.08, 31.74 and 32.56 minutes, respectively (Figure S2, A and B). Again, mass spectra confirmed the identities of these PUFAs (Figure S2, C-E). Other prostanoids i.e. 6-keto-PGF_1α_ and PGD_2_ were not detected in *P. monodon* extracts from stage I-III shrimp ovaries, hepatopancreases, lymphoid organs or hemolymph.

### Prostaglandin synthase activity in *P. monodon* ovarian tissue

**Figure 6:**
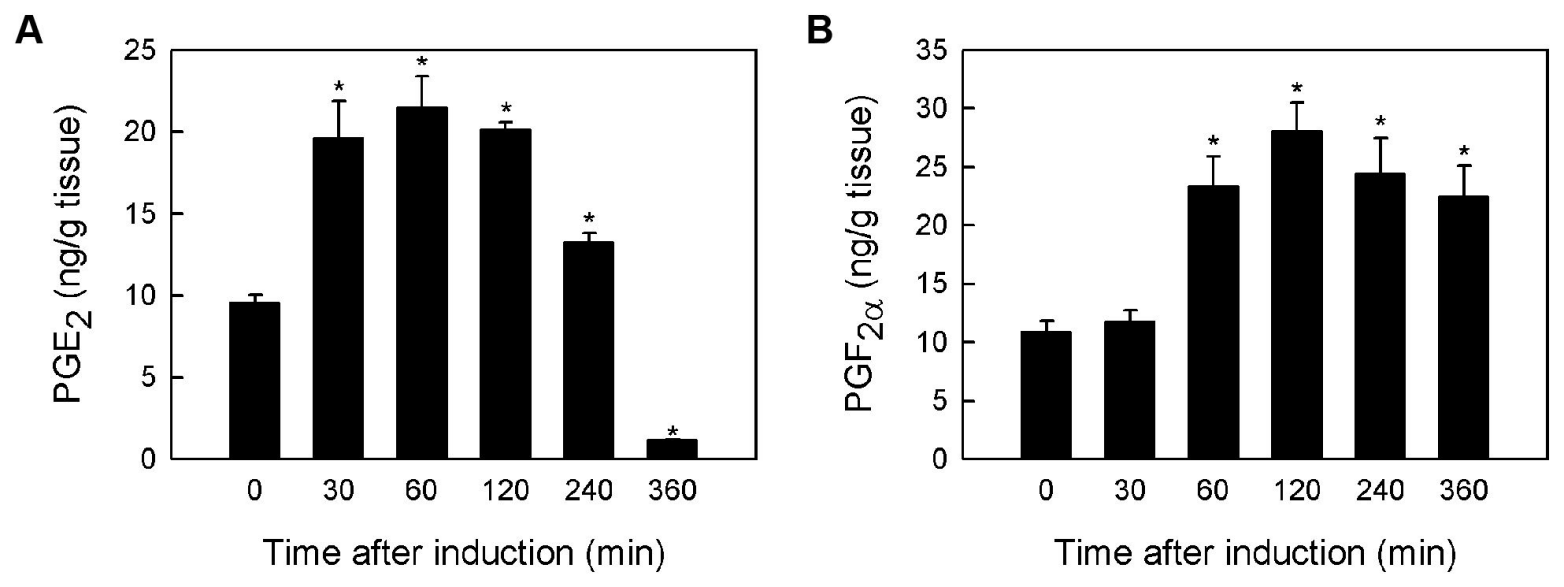
*In vitro* prostaglandin synthase activity assay using shrimp ovary homogenates. Shrimp ovary homogenates were incubated with 25 µM AA at 28 °C and collected at different time points (0, 30, 60, 120, 240 and 360 min). The homogenates were spun down and concentrations of PGE_2_ (A) and PGF_2α_ (B) in the homogenate supernatant were estimated using EIA. The experiment was performed in triplicate and error bars indicate the standard deviation from the means. Asterisk indicates significant difference between the prostaglandin concentration at 0 h and the marked time point (*P*<0.05).

The identification of *PmPGES* and *PmPGFS* genes and their corresponding products led us to speculate that prostaglandin synthase activity could be present in shrimp ovaries. To test this hypothesis, *in vitro* prostaglandin synthase activity assay was performed by incubating shrimp ovary homogenates with 25 µM AA at 28 °C, 200 rpm. The samples were subsequently collected at different time points to monitor prostaglandin production. Prior to the treatment, the basal concentration of PGE_2_ in shrimp ovary homogenates was 9.6 ng/g tissue. After 30 minutes of incubation with AA, the PGE_2_ concentration increased to 19.6 ng/g tissue and remained at this level before declining after 120 minutes of treatment (Figure 6A). Similarly, the concentration of PGF_2α_ increased from the basal level of 10.9 ng/g tissue to 23.3 ng/g tissue after 60 minutes of incubation with AA and remained at the same level from 60 to 360 minutes after the treatment (Figure 6B). Together, these results suggest that the prostaglandin synthase activity is present in *P. monodon* ovary homogenates.

### Role of prostaglandin biosynthesis in shrimp ovarian development

**Figure 7:**
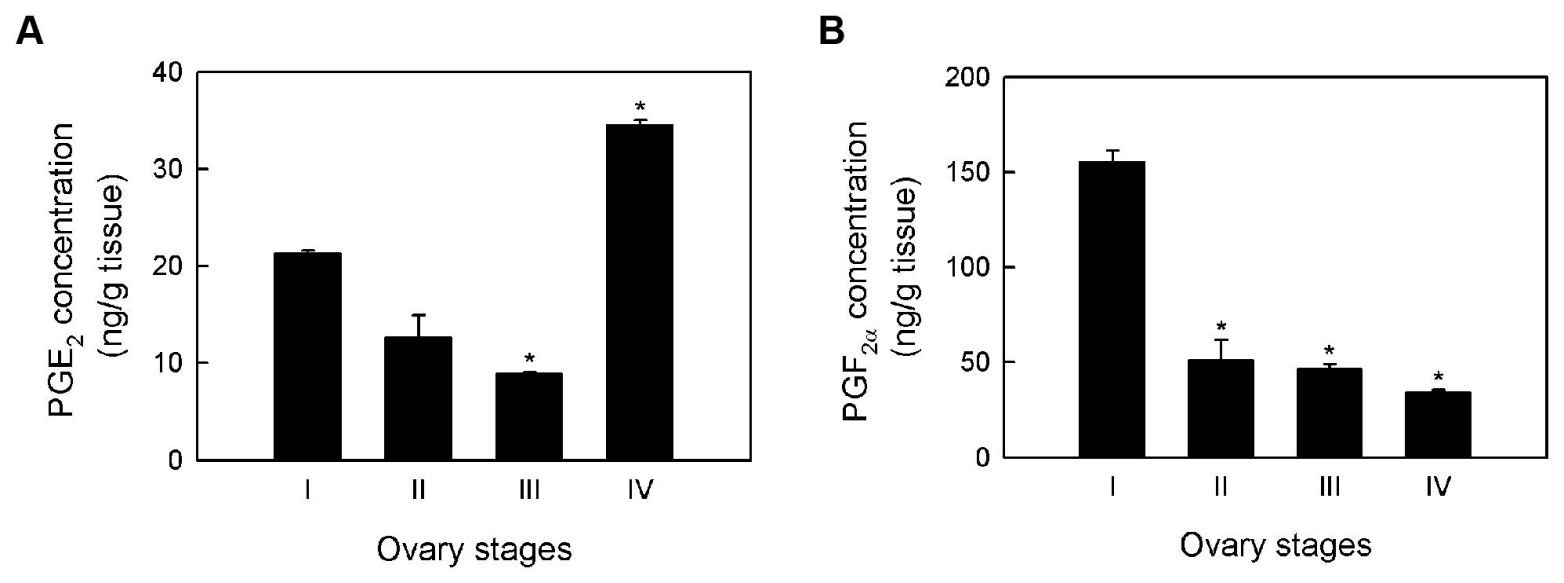
PGE_2_ and PGF_2α_ concentrations in each ovary stage. Shrimp ovaries from each stage were pooled together based on the GSI value (N = 3 for each ovary stage) and homogenized. The amounts of (A) PGE_2_ and (B) PGF_2α_ were determined using enzyme immunoassay. Error bars indicate the standard deviation from the means. These graphs are representatives of two independent experiments that yielded similar results.

The correlation between the amounts of prostaglandin and ovarian maturation in *P. paeninsulanus* [43], *M. japonicus* [10] and *O. senex senex* [20] led to the hypothesis that prostaglandins affect female reproductive development in crustaceans. To assess the possible role of prostaglandin biosynthesis in *P. monodon* ovarian maturation, we estimated the concentrations of PGE_2_ and PGF_2α_ in different stages of shrimp ovaries. When compared with stage I ovaries, we observed that the PGE_2_ concentrations were lower in ovaries stage II and III and higher in ovaries stage IV (Figure 7A). On the other hand, the PGF_2α_ concentrations were highest in stage I ovaries and steadily decreased as the ovaries matured (Figure 7B).

**Figure 8:**
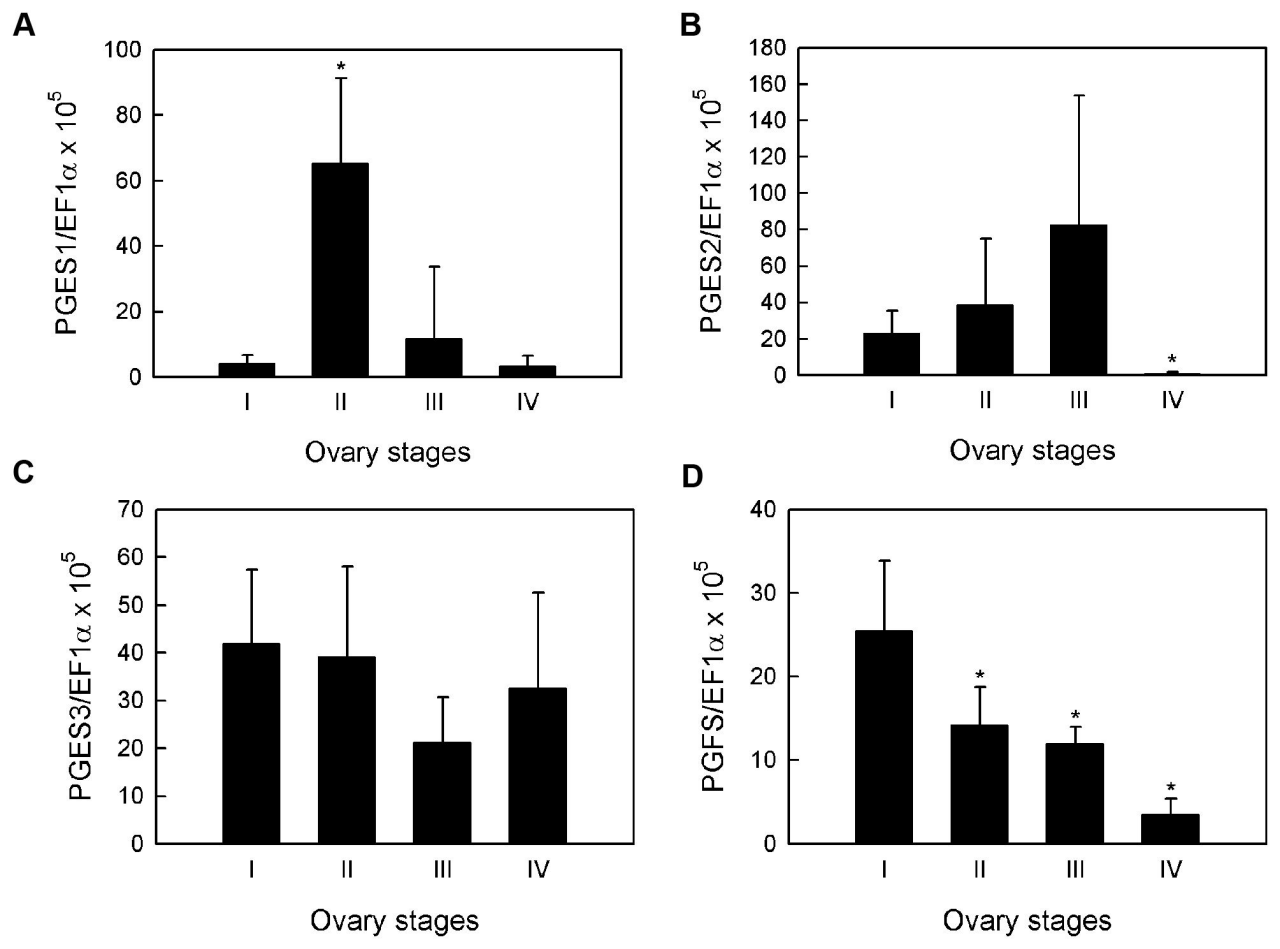
Relative expression levels of *PmPGES* and *PmPGFS* genes in each ovary stage. Wild broodstock from the Andaman Sea (N=27) were captured and dissected to obtain ovary samples used in the real-time PCR analysis. Each graph represents the average copy number of prostaglandin biosynthesis gene transcripts normalized against *EF1α* in each ovary stage. (A) *PmPGES1*, (B) *PmPGES2*, (C) *PmPGES3* and (D) *PmPGFS*. Error bars show standard deviations and asterisks indicate significant changes between stages (p < 0.05).

To correlate the mRNA expression levels of prostaglandin biosynthesis genes with different ovarian maturation stages, quantitative real-time PCR analysis was performed on ovaries of 27 wild-caught broodstock from the Andaman Sea (Table 2). Four sets of primers for *PmPGES1*, *PmPGES2*, *PmPGES3* and *PmPGFS* genes were used in this study (Table 3). To determine whether the gene was up- or down-regulated at a certain developmental stage, mRNA expression levels were compared with that of stage I ovaries, which was taken as a baseline. It was observed that all three *PmPGES* isoforms displayed different expression profiles during the development of ovaries. *PmPGES1* was up-regulated 16-fold in stage II ovaries, then continued to decrease until it reached baseline in stage IV (Figure 8A). For *PmPGES2*, the change in expression from stage I to stage III ovaries was not significant. However, a significant 27-fold down-regulation of this transcript was observed in stage IV ovaries (Figure 8B). The *PmPGES3* expression level did not change significantly throughout ovarian development (Figure 8C). Lastly, the amount of *PmPGFS* gene transcripts steadily decreased as the ovary maturation progressed from stage I to stage IV (Figure 8D).

**Table 2:** Lists of ovary maturation stage, the number of shrimp (N), average body weight, body length, ovary weight and GSI of the wild *P. monodon* samples used in real-time PCR analysis.

**Ovary stages**	**N**	**Body Weight (g)**	**Body length (cm)**	**Ovary weight (g)**	**GSI**
1	7	256.59±26.51	30.00±0.94	4.33±0.72	1.79±0.10
2	7	250.47±41.40	30.50±2.65	6.19±1.26	2.47±0.21
3	7	239.36±44.01	30.00±3.55	12.29±2.79	5.12±0.45
4	6	233.46±33.47	29.33±1.54	15.56±2.58	6.95±0.37

**Table 3:** Primer sequences used in quantitative real-time PCR analysis.

**Genes**	**Primers**	**Sequence**
*PGES1*	PGES1-RT-F	CAAGAAGGTATTCGCCAACC
	PGES1-RT-R	CCGTTGCTCCTCAGGTACATA
*PGES2*	PGES2-RT-F	GGGAAGCATCCACCTGGACGTTCC
	PGES2-RT-R	GGTGCCGTCTCTTCAGGTTCTTGCC
*PGES3*	PGES3-RT-F	GACTGCAAATCTCCCACCAT
	PGES3-RT-R	ACTTTGAGCCAGTGCTGCTT
*PGFS*	PGFS-RT-F	GGAGAAGTAATGCAGGCTGT
	PGFS-RT-R	GCCAGGTCTCAATGTAATCC

## Discussion

In this study, the characterization of the *P. monodon* prostanoid pathway reveals that *P. monodon* contains the same types and number of prostaglandin synthase isoforms as those found in mammals (Fig, 1-4; Table 1). In addition to the three *PmPGES* isoforms shown in the Results section, *P. monodon* also encodes two isoforms of *prostaglandin D synthase* that matched the *glutathione-dependent prostaglandin D synthase* and *hematopoietic prostaglandin D synthase* genes originally identified in mammals. Interestingly, the proposed prostanoid pathway in another crustacean *Daphnia pulex* contains only one isoform of each prostanoid biosynthesis gene [22], suggesting that the organization of the *P. monodon* prostanoid pathway is more conserved with those in mammals, or other divergent prostanoid biosynthetic genes are present in *D. pulex* that are not annotated. To compare if the *P. monodon* prostanoid protein sequence is more closely related to its homologs in *D. pulex* or mammals, phylogenetic analysis of PGES1 was performed, revealing that PmPGES1 is more closely related to its crustacean homologs, including *D. pulex*, than to its mammalian homologs (Figure S3).

PUFAs and PGE_2_ have previously been identified in domesticated *P. monodon*, although it was not clear whether these molecules were synthesized *de novo* as the animals had been fed with high-PUFA diets [13,21]. In this study, PGF_2α_ was detected by RP-HPLC and mass spectrometry analysis in ovaries of wild-caught *P. monodon*, suggesting that these molecules occurred naturally in wild population consistent with de novo synthesis and were not the result of specific diets or rearing conditions. Although *PmPGDS* and *PmTBXAS* genes are present in *P. monodon* (Table 1), we were unable to detect the corresponding prostanoid products PGD_2_, PGF_1α_ and TBX_2_ which have previously been identified in other arthropods [1,20,44,45]. Therefore, these prostanoids may be present in small amounts in *P. monodon*.

To provide further proof of a functioning prostanoid synthetic pathway, we assessed the prostaglandin synthase activity in penaeid shrimp using an *in vitro* activity assay to monitor PGE_2_ and PGF_2α_ biosynthesis in *P. monodon* [46]. After shrimp ovary homogenates were incubated with AA, the PGE_2_ and PGF_2α_ concentrations increased significantly when compared to the basal concentrations (Figure 6). In particular, the rise and fall of PGE_2_ concentrations observed in this study is similar to the study performed on *A. americanum* [47]. As the PGE_2_ biosynthesis activities have already been established in the Tobacco hornworm *Manduca sexta* [48], the blood sucking bug *Triatoma infestans* [49], the firebrat *Thermobia domestica* [50] and the cricket *Teleogryllus commodus* [51], we propose that the PGE_2_ biosynthesis is conserved among different arthropods.

Once the prostanoid pathway had been established in *P. monodon*, we examined whether there is a correlation between prostanoid biosynthesis and shrimp ovary development. In wild *P. monodon* broodstock, the concentrations of PGE_2_ decreased from stage I to stage III, but abruptly increased in stage IV ovaries (Figure 7A). This is inconsistent with the trend found in domesticated shrimp, in which the amounts of PGE_2_ gradually increased during ovary development [21]. The discrepancy between the two studies may be the result of different shrimp genetic background and/or dietary intake. Interestingly, the PGE_2_ concentrations were highest at stage IV ovaries in both wild and domesticated shrimp, but the significance of this observation has yet to be explored. When the gene expression of each *PmPGES* isoform was compared to the concentration of PGE_2_ found in each ovary stage, we observed no correlation between the two parameters. Furthermore, the lowest amount of *PmPGES* transcripts was found in stage IV ovaries, making it unlikely that the PGE_2_ biosynthesis is regulated at the transcriptional level in this organ.

Unlike PGE_2_, the amount of PGF_2α_ steadily decreased with increasing ovary stages, which followed the same trend observed in *M. japonicus* [10]. Similarly, the *PmPGFS* gene expression also decreased as the ovaries matured, making *PmPGFS* gene expression and PGF_2α_ concentration inversely correlated with shrimp ovarian development. Together, our findings suggest that lowered *PGFS* gene expression resulted in decreasing concentrations of PGF_2α_ during shrimp ovarian maturation process. Therefore, we propose that the PGF_2α_ biosynthesis may be involved in the *P. monodon* ovarian development.

**Figure 9:**
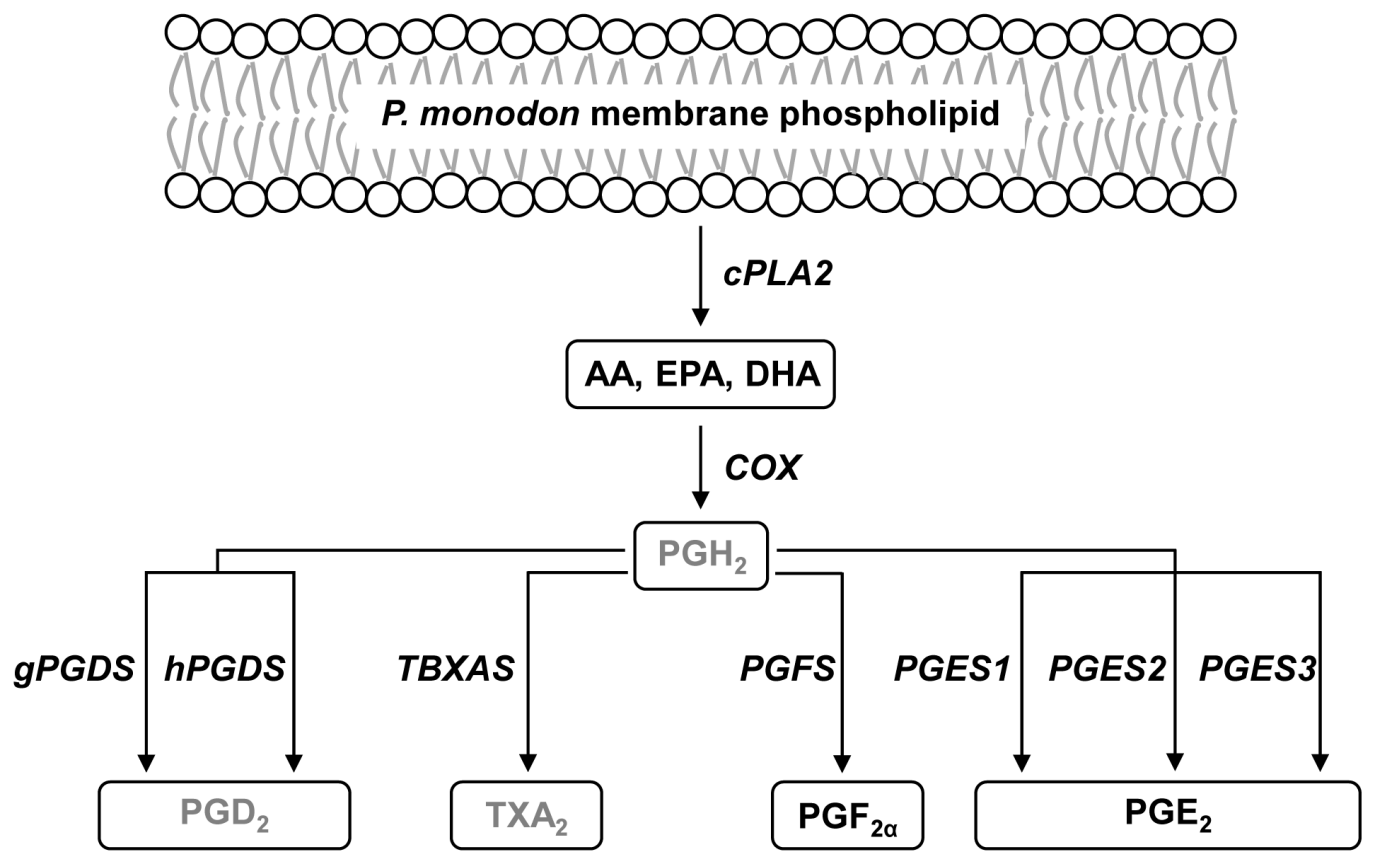
The proposed prostanoid biosynthesis pathway in *P. monodon*. PUFAs, prostaglandins and full-length prostanoid biosynthesis genes identified in this study (black) were used to outline the *P. monodon* prostanoid pathway based on the previously published pathway in mammals. Prostanoids that have yet to be identified in *P. monodon* are shown in gray.

In conclusion, a prostanoid pathway in *P. monodon* is proposed based on the identification of nine prostanoid biosynthesis genes, two prostaglandins and three prostanoid precursors (Figure 9). In addition, PGF_2α_ biosynthesis may play an important role in *P. monodon* ovarian maturation because PGF_2α_ concentration and *PmPGFS* gene expression declined as the ovarian development progressed. Collectively, our knowledge of the *P. monodon* prostanoid pathway may lead to future applications in the black tiger shrimp aquaculture industry. More importantly, the identification of the *P. monodon* prostanoid biosynthesis genes also suggests the conservation of the prostanoid pathway between marine crustaceans and mammals.

## Materials and Methods

### Collection of shrimp samples

Wild female *P. monodon* broodstock (n = 25) were collected from the Andaman Sea, Thailand. The weight and length of broodstock were measured and recorded prior to dissection. Shrimp ovaries were dissected, weighed and either subjected to metabolite extraction or flash frozen in liquid nitrogen and stored at -80 °C for RNA extraction. The gonadosomatic index (GSI) of each shrimp was calculated using the following equation: ovarian weight/body weight x 100. Ovarian developmental stages were assigned according to GSI, separating the broodstock into stage I (GSI < 1.5), stage II (GSI = 2-4), stage III (GSI > 4-6) and stage IV (GSI > 6) [21].

### Extraction of prostaglandins from shrimp tissues

Tissue homogenates underwent extraction twice with an equal volume of ethyl acetate. The solvent phases were then pooled together and dried under vacuum. Afterward, the dried crude extract was dissolved in 5% methanol. The solution was filtered to remove insoluble material before being loaded onto a 6-ml C18 SPE cartridge (VertiPak^TM^, Vertical Chromatography, Co., Ltd., Thailand), which was previously washed with 10 ml methanol and 10 ml water. Columns were then washed with 10 ml water, 4 ml hexane, and again with 10 ml water, before being eluted with 10 ml ethyl acetate. The eluate was then evaporated and dissolved in ethanol for subsequent HPLC analysis.

### Identification of PUFAs and prostaglandins by RP-HPLC and mass spectrometry

Shrimp tissue extracts were separated by RP-HPLC using an Acclaim^®^ 120 C18 column (3 µm, 4.6 mm x 150 mm; DIONEX Ltd., Surrey, UK) and a gradient mobile system consisting of acetonitrile (ACN): water: acetic acid (30:70:0.01) to 100% ACN in 35 min, with the flow rate of 0.8 ml/min. Resulting peaks were detected using the DIONEX Ultimate 3000 diode-array detector (DIONEX Ltd.). For RP-HPLC/MS analysis, samples were analyzed using the Agilent 1200 series LC system (Agilent Technologies Inc., Santa Clara, CA, USA) coupled with the micrOTOF mass spectrometer and operated with HyStar version 3.2 (Bruker Daltonics Inc., Billerica, MA, USA).

### RNA extraction and cDNA synthesis

Shrimp organs were homogenized and subjected to total RNA extraction using the Trizol reagent (Invitrogen, California, USA). mRNA was purified from total RNA using the Oligotex mRNA Mini Kit (QIAGEN, Maryland, USA). First strand cDNA was synthesized using the RevertAid^TM^ First Strand cDNA Synthesis Kit with oligo (dT)_18_ primer (Fermentas, Maryland, USA) according to the manufacturer’s instructions.

### Gene amplification

Initial PCR fragments were obtained using primers based on short gene sequences from The Black Tiger Shrimp EST Project at http://pmonodon.biotec.or.th [26] and the Marine Genomics Project at http://www.marinegenomics.org [24]. 5′- and 3′-RACE-PCR were performed using the Advantage^TM^ 2 PCR kit (Clontech, California, USA) and the SmartTM RACE cDNA amplification kit (Clontech). All PCR products were cloned into the pTZ57R/T vector (Fermentas), transformed into DH5α *E. coli*, and submitted for DNA sequencing (1^st^-BASE, Malaysia). Identities of the obtained cDNA sequences were verified by TBLASTX analysis [52]. Multiple sequence alignment of prostaglandin biosynthesis genes were performed using CLUSTALX [53]. Rooted phylogenetic tree with branch length were performed using CLUSTALW [53]. Protein domains were predicted using Conserved Domain Architecture Retrieval Tool (CDART - http://www.ncbi.nlm.nih.gov/Structure/lexington/lexington.cgi) [54].

### Quantitative real-time PCR analysis

Quantitative real-time PCR analysis was performed using the SsoFast^TM^ EvaGreen^®^ Supermix (Bio-Rad, California, USA) according to the manufacturer’s instructions. Amounts of prostanoid biosynthesis gene transcripts relative to that of the house-keeping gene elongation factor 1α (EF1α) were obtained using the standard curve method [55]. The specificity of the PCR product was confirmed by agarose gel electrophoresis and melting curve analysis performed from 55 °C to 95 °C with a continuous fluorescent reading at 0.5 °C increments.

### Identification of shrimp PGE_2_ and PGF_2α_ by enzyme immunoassay (EIA)

Stage IV shrimp ovaries were harvested and homogenized in Hank’s Balanced Salt Solution (HBSS) with an osmolarity of 720 mmol/kg (Sigma-Aldrich Inc., Missouri, USA). For the *in vitro* PGE_2_ synthesis assay, ovary homogenates were incubated in a rotary shaker with 25 µM AA at 28 °C, 200 rpm and collected at 0, 30, 60, 120, 240, and 360 minutes post-incubation. The homogenates were centrifuged at 12,000 x g for 2 min at 4 °C and the amounts of PGE_2_ and PGF_2α_ in the supernatant were estimated using the prostaglandin E_2_ EIA kit – Monoclonal and prostaglandin F_2α_ EIA kit (Cayman Chemical, Michigan, USA).

### Statistical analysis

Statistical significant was assessed in this study using the T-test with two samples assuming equal variances (*P*<0.05).

## Supporting Information

Figure S1 **Schematic representation of domain types and positions on each putative *P. monodon* prostanoid biosynthesis enzyme.** Prostanoid biosynthesis gene sequences were submitted for the CDART analysis for domain prediction. Solid lines represent the total length of each predicted protein, while ovals and squares denote the conserved domains. C2 domain was first identified in phosphokinase C. TRX is thioredoxin-like superfamily domain. GST_C is glutathione transferase family, C-terminal alpha helical domain. MAPEG is Membrane-Associated Protein involved in Eicosanoid and Glutathione metabolism domain. EGF_CA is calcium-binding, EGF-like domain. An_peroxidase-like is animal heme peroxidases and related protein.
(TIF)

Figure S2 **HPLC analysis and mass spectra of prostanoid precursors in shrimp ovary extract.** Ovaries from 5 wild broodstock were homogenized in HBSS, pooled together, and incubated at 28 °C, 200 rpm for 1 h. The homogenate was extracted as described in materials and methods. The extract was then subjected to analysis by RP-HPLC and mass spectrometry. RP-HPLC elution profiles of commercially available prostanoid standards (A) and prostanoid precursors found in ovary homogenate (B) was obtained at 200 nm wavelength. Subsequent mass spectrometry analysis of prostanoid precursors in ovary homogenate revealed the mass spectra of EPA (C), DHA (D) and AA (E).
(TIF)

Figure S3 **Phylogenetic trees constructed from *PmPGES1* and related sequences from vertebrates and invertebrates.** Predicted amino acid sequences from various organisms were obtained from GenBank and the Marine Genomics Project. Sequences were aligned using CLUSTALW multiple sequence alignment program and the rooted phylogenetic tree with branch length (UPGMA) were constructed.
(TIF)
