# Supplementary figures and images for: Insights into the Prostanoid Pathway in the Ovary Development of the Penaeid Shrimp Penaeus monodon
Source: PLoS One. 2013 Oct 8;8(10):e76934. doi: 10.1371/journal.pone.0076934 (PMC3792876; doi:10.1371/journal.pone.0076934)

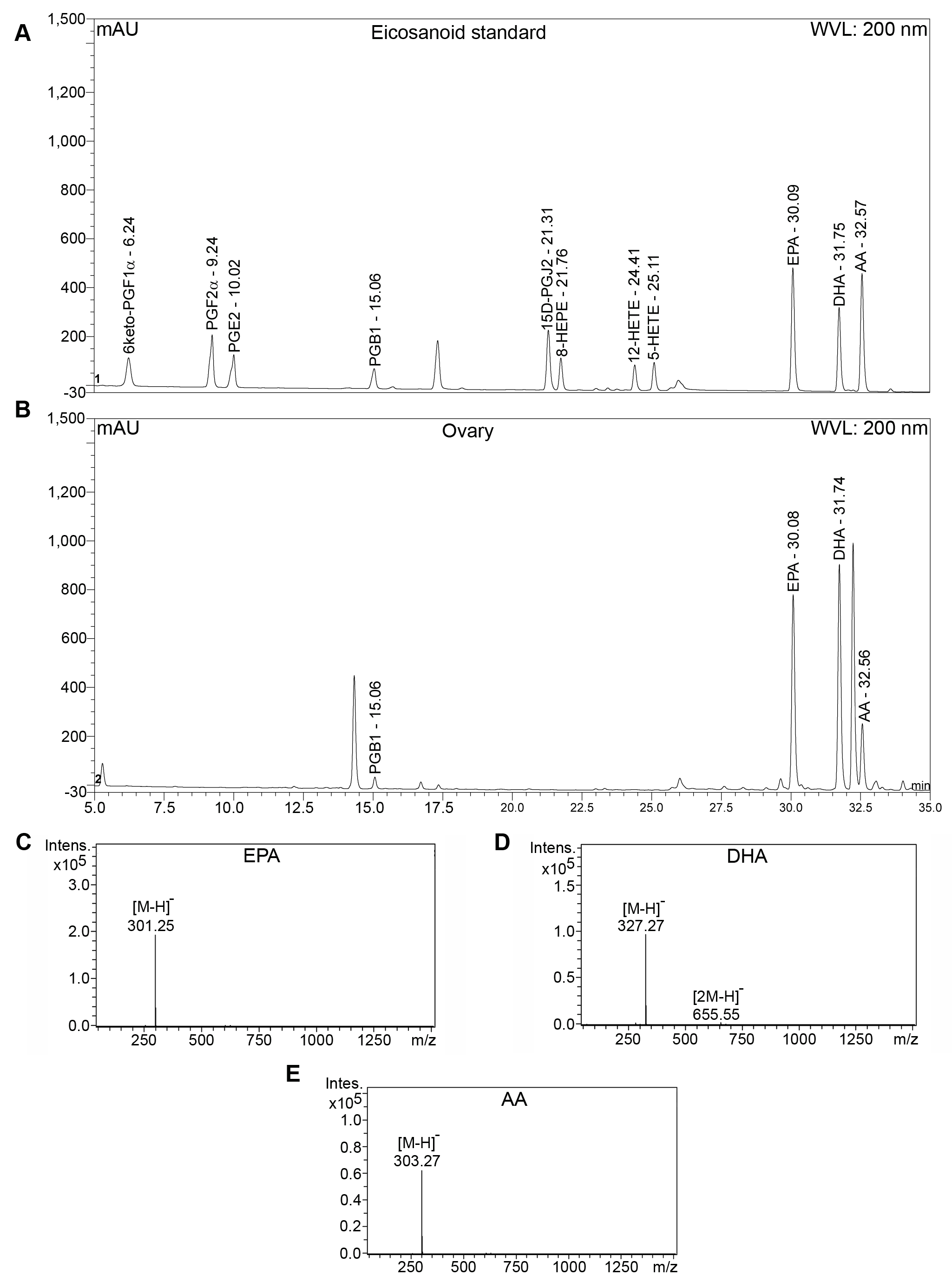

Supplement: Figure S1 — Schematic representation of domain types and positions on each putative P. monodon prostanoid biosynthesis enzyme. Prostanoid biosynthesis gene sequences were submitted for the CDART analysis for domain prediction. Solid lines represent the total length of each predicted protein, while ovals and squares denote the conserved domains. C2 domain was first identified in phosphokinase C. TRX is thioredoxin-like superfamily domain. GST_C is glutathione transferase family, C-terminal alpha helical domain. MAPEG is Membrane-Associated Protein involved in Eicosanoid and Glutathione metabolism domain. EGF_CA is calcium-binding, EGF-like domain. An_peroxidase-like is animal heme peroxidases and related protein. (TIF) [file pone.0076934.s001.tif]

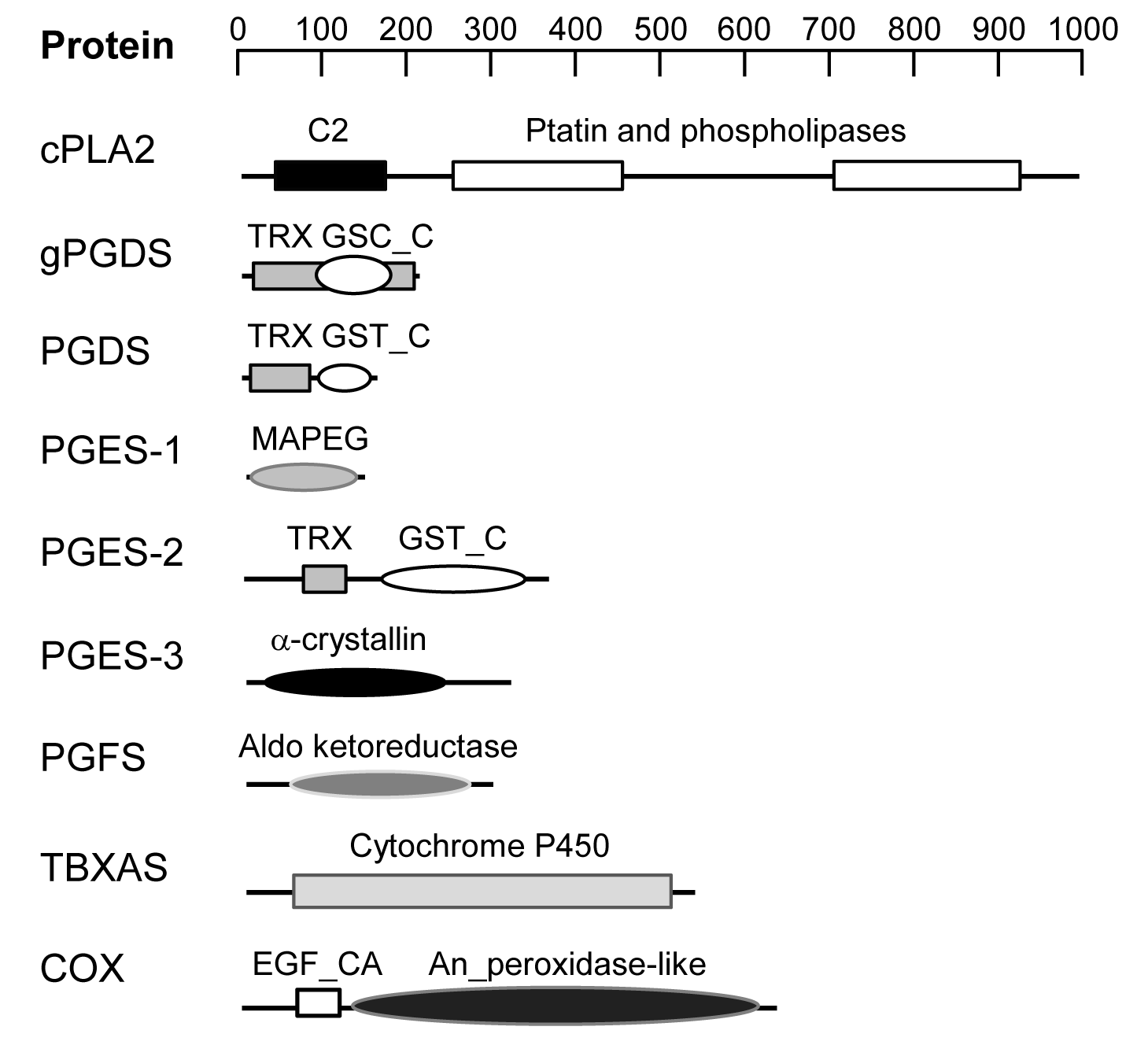

Supplement: Figure S2 — HPLC analysis and mass spectra of prostanoid precursors in shrimp ovary extract. Ovaries from 5 wild broodstock were homogenized in HBSS, pooled together, and incubated at 28 °C, 200 rpm for 1 h. The homogenate was extracted as described in materials and methods. The extract was then subjected to analysis by RP-HPLC and mass spectrometry. RP-HPLC elution profiles of commercially available prostanoid standards (A) and prostanoid precursors found in ovary homogenate (B) was obtained at 200 nm wavelength. Subsequent mass spectrometry analysis of prostanoid precursors in ovary homogenate revealed the mass spectra of EPA (C), DHA (D) and AA (E). (TIF) [file pone.0076934.s002.tif]

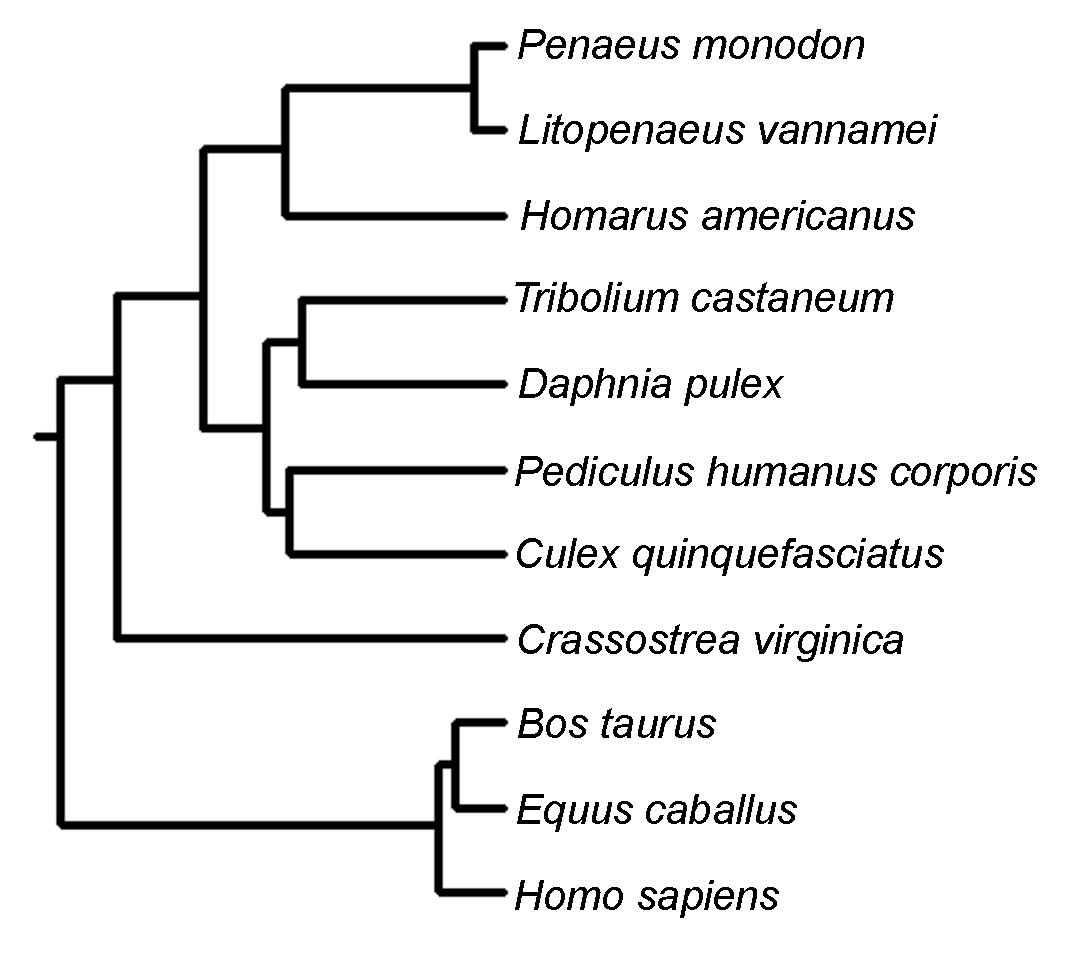

Supplement: Figure S3 — Phylogenetic trees constructed from PmPGES1 and related sequences from vertebrates and invertebrates. Predicted amino acid sequences from various organisms were obtained from GenBank and the Marine Genomics Project. Sequences were aligned using CLUSTALW multiple sequence alignment program and the rooted phylogenetic tree with branch length (UPGMA) were constructed. (TIF) [file pone.0076934.s003.tif]
